# An ancient examination in the face of a modern pandemic: systematic review of major clinicopathological autopsy findings

**DOI:** 10.1590/1806-9282.20210098

**Published:** 2022-08-19

**Authors:** Miguel Augusto Martins Pereira, Lucas Natã Lessa e Silva, Matheus Pires de Almeida Lessa, Jéssica Cunha, Ana Caroline Siquara de Souza, Luciana Pantaleão

**Affiliations:** 1 Universidade Federal Fluminense, Fundação de Amparo à Pesquisa do Estado do Rio de Janeiro – Niterói (RJ), Brazil.; 2 Universidade Federal Fluminense – Niterói (RJ), Brazil.; 3 Universidade Federal Fluminense, Departamento de Patologia – Niterói (RJ), Brazil.

## INTRODUCTION

Autopsy consists of the examination of a corpse to determine the time and cause of death, as well as to evaluate any disease or injury that may have been present. Initially, autopsies functioned primarily as an anatomical analysis, but in the eighteenth century, they have taken on an investigative function through the study of pathological findings^1^.

Due to new imaging methods, the reluctance of families, and new regulations, the performance of autopsy examinations started to decline in the 1980’s. However, autopsies still play an important role in learning and reducing the rate of diagnostic errors^2^. Autopsies are fundamental to modern medicine, as in the evaluation of clinical procedures and medical education. Furthermore, in view of the recent circumstances of the coronavirus disease 2019 (COVID-19) pandemic, they are gaining more prominence for the purposes of studying this disease responsible for great socioeconomic and world health damage, whose pathophysiology is still poorly understood^3^.

Thus, the purpose of this review was to clarify the role of autopsy in the context of the COVID-19 pandemic, as well as to present the main findings in autopsy examination of patients diagnosed with COVID-19.

## METHODS

This review was conducted by two independent researchers, using the SciELO, PubMed, LiLacs, and Scopus databases. The following descriptors were chosen: “Coronavirus,” “SARS-CoV-2,” and “Autopsy.” The filters used were as follows: Language English; article type: Classical Article, Clinical Study, Journal Article, Multicenter Study; within the past 1 year; and in English or Portuguese. Inclusion criteria were as follows: articles containing information on findings in autopsy examination of patients with a confirmed diagnosis of COVID-19. Exclusion criteria were as follows: articles not published in English or Portuguese; review articles, case report studies, and case series.

**Figure 1.**
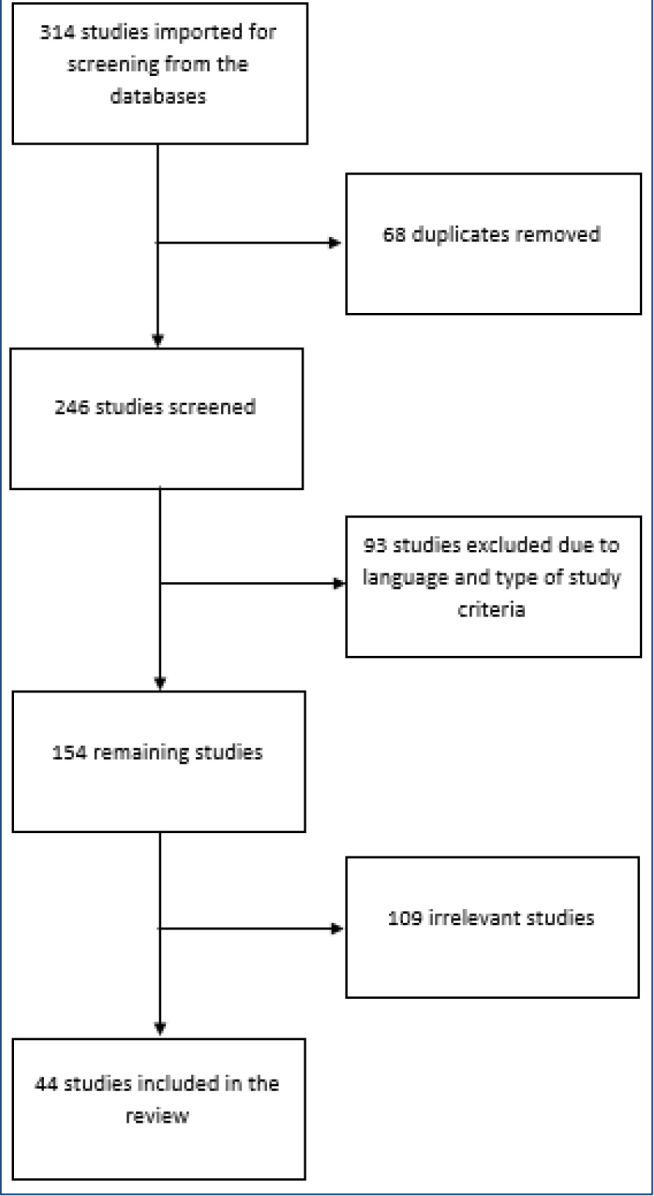
Linear flowchart of the screening and selection of studies.

**Figure 2.**
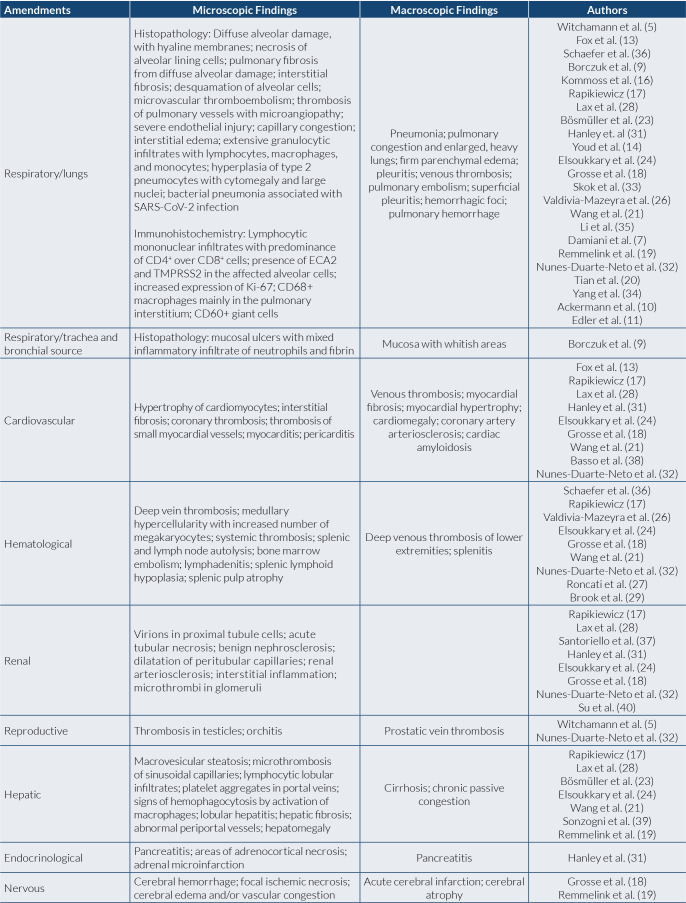
Main Systemic findings of autopsy studies.

The selection occurred in three stages (Figure 1). First, studies were identified from the PubMed search string ((((Covid-19[Title/Abstract]) OR (Sars-Cov-2[Title/Abstract])) AND (Autopsy[Title/Abstract])) AND (English[Language])); and in Scopus, -(-TITLE-ABS-KEY-(-covid-19) -OR -TITLE-ABS-KEY (sars-cov-2) AND TITLE-ABS-KEY (autopsy)) -AND.- DOCTYPE (-ar -) -AND. PUBYEAR. -> 2018. -AND. -(-LIMIT-TO- (-LANGUAGE -, - “English”-) -). In the second stage, articles were excluded according to the type of study and language. In the last stage, the titles and abstracts of the remaining studies were read and those with nonconsistent themes were excluded. Finally, the remaining studies were included in this review (Figure 2).

## DISCUSSION

### Autopsy in the history of medicine

In prehistoric times, Inuit and Australian Aborigines studied mammalian anatomy by hunting large animals. However, it was noted in Greece history that autopsy was initiated to be applied in the medical sciences. This legacy was almost lost during the Middle Ages due to positions contrary to autopsies^1^.

The educational and scientific changes that occurred with the industrial revolution led to the appearance of dissecting rooms in most large hospitals, where anatomopathology was introduced as the basis for diagnosis and nosology. The performance of autopsies increased considerably when Cabot proved that they could detect misdiagnoses and the Flexner Report criticized the state of medical education in the United States. However, the occurrence of autopsies decreased again from the 1980’s^1^,^2^.

In contemporary times, many medical specialties are related to autopsy, such as forensic medicine and cardiology. In addition, autopsy has developed into several specialties, such as radiological, microbiological, and molecular autopsy^2^.

### The role of autopsy in COVID-19

#### Respiratory changes

Severe acute respiratory syndrome coronavirus 2 (SARS-CoV-2) infection in the respiratory tract is caused by the surface protein S, which binds to angiotensin-converting enzyme 2 (ACE2), expressed in cells of the nasal epithelium and in large quantities in type 2 pneumocytes in the lower respiratory tract. Through immunohistochemical examination, the presence of ACE2 was confirmed in alveolar cells damaged by SARS-CoV-2 infection^4^.

At the macroscopic level, several studies have described congested and heavy lungs, with their surface exhibiting pleuritis and a distinctive irregular mosaic pattern of pale areas alternating with purplish and dark hypercapillary areas, which are slightly protuberant, such that the pattern is visible on the cut surfaces^3,5,6,7,8,9^. In addition, the lung tissue is both firm and friable.

Microscopy frequently indicates the presence of diffuse alveolar damage, both exudative and proliferative, which is a nonspecific finding. There is also alveolar inflammation, with the presence of hyaline membranes, hyperplasia of type 2 pneumocytes, microvascular thromboembolism, capillary congestion, interstitial edema, intra-alveolar fibrin deposition, interstitial fibroblasts, and squamous metaplasia in the more advanced cases^5,6^. According to Fox et al., the inflammatory infiltrate was composed of a mixture of CD4+ and CD8+ T lymphocytes, located predominantly in the interstitium and around the bronchioles and blood vessels^9^.

Borczuk et al. verified the presence of focal white spots on the mucosa of the upper and middle airways, hyperemic pharyngeal mucosa, and mixed inflammatory infiltrate, predominantly lymphocytic, with the presence of fibrin and ulcerations. However, they highlighted that there was no significant evidence correlating these findings with intubation and bacterial or fungal pneumonia^5^.

#### Cardiovascular alterations

In some autopsies, the presence of elevated cardiac enzymes (troponin T and/or B-type natriuretic propeptide amino terminal fraction) was observed. The increase in troponin three days before the death of some patients corroborates a possible association between troponin elevation and mortality. This increase may have several causes, such as thrombosis of the microvasculature and cardiac veins^9–11^.

A significant finding was right ventricular dilatation from elevated brain natriuretic peptide, resulting from pulmonary hypertension due to damage generated in the pulmonary vessels by the disease^9,10^.

Tian et al. and Grosse et al. found important cardiovascular changes, such as myocardial hypertrophy, acute myocardial infarction, focal myocardial fibrosis, and coronary atherosclerosis, which may be related to preexisting diseases. Also, we observed lymphocytic inflammatory infiltrate, although not significant, associated with damage to cardiomyocytes, with no evidence of viral myocarditis and no characteristics of viral cytopathic effect observed. These changes, therefore, may be secondary or related to underlying diseases^9–13^.

As the polymerase chain reaction only detects the residual viral genome, it is unknown whether viral particles in the cardiac cells correspond to active viral replication or a previous infection without clinical relevance^12^. In contrast, there are other potential mechanisms of myocardial injury, such as severe respiratory infection with hypoxia, sepsis, systemic inflammation, pulmonary thrombosis and thromboembolism, cardiac adrenergic hyperstimulation during cytokine release syndrome, and myocarditis^11,13^.

A numerically significant finding of COVID-19-positive individuals was massive cardiac amyloidosis, assuming that these patients died from cardiac decompensation^14^.

There are indications that fulminant myocarditis can occur in SARS-CoV-2 infection, likely contributing to the morbidity of COVID-19. However, there are cases of “acute cardiac injury” in patients that do not necessarily translate into myocarditis or acute myocardial ischemia and no significant lymphocytic inflammatory infiltrates were found, highlighting the need for further studies on the cardiac impacts of SARS-CoV-2. These cardiomyopathies were also associated with metabolic disorders, such as severe metabolic and respiratory acidosis, recurrent in patients with COVID-19, which is another variable that should be analyzed in their pathogenesis^9–13^.

Megakaryocytes were also found, with higher levels in the thrombi and vascular beds, cardiac tissue, and bone marrow. The morphology of these cells suggests active platelet production. This could contribute significantly to thrombosis, which is related to multiple organ failure, severe hypoxia, and death in COVID-19 patients^9^.

#### Hematological alterations

Hematological alterations are closely related to the pathogenesis of COVID-19. There is evidence that the presence of microthrombi is associated with lesions present in several organs besides the lungs. Studies point out that under certain inflammatory conditions, there is an attempt to contain pathogens through the aggregation of platelets, neutrophils, and the coagulation cascade, a process called immunothrombosis. Nicolai et al. confirmed the presence of neutrophils embedded in fibrin clots in the microthrombi formed in this process, in addition to the existence of an increased number of thrombi containing granulocytes in autopsies of COVID-19 patients when compared with the lungs of patients who died of nonpulmonary diseases^15^. Elevation in fibrin degradation product (D-dimer) corroborates the association between a procoagulant state and disease severity, as does the histopathological evidence of microvascular thrombosis in the affected organs^3,15,16^.

Several studies have demonstrated the presence of platelet-rich thrombi in the pulmonary, renal, cardiac, and hepatic microvasculature, which was also observed in the presence of megakaryocytes, bone marrow, microvasculature of the heart, and glomeruli, which was higher than usual in the lungs^3,10,17,18^. This elevation is possibly due to the state of hypercoagulability caused by severe cases of the disease^17^. Thrombosis has been found in several organs at different stages of the disease course, even with complete anticoagulation treatment, suggesting its great relevance in the disease process^10,15^.

In macroscopic analysis of the lymph nodes, an increase in their structure was noted, with lymphocyte depletion and the absence of germinal centers^20^. The splenic white pulp was atrophied due to lymphocyte depletion^20,21^. Brook et al. also observed an increase in the red pulp and the presence of irregular necrosis in the spleen or large areas of infarction, which they related to be possibly due to shock^21^.

#### Renal changes

SARS-CoV-2 viral RNA at high titers was detected in the kidneys of some patients who died from COVID-19^3^. On histopathological examination, renal signs of shock were found in most autopsies, such as diffuse acute tubular necrosis with enlarged tubular lumen, flattened tubular epithelium, and interstitial edema. In addition, small fibrin thrombi were found in the glomerular capillaries^8,20,22^.

Similar to the pulmonary tissue, chronic inflammatory infiltrate was observed in areas with interstitial fibrosis and tubular atrophy. In transmission electron microscopy, podocytes with prominent activation containing several vesicles with virus-like particles in the cytoplasm were visualized, relating to SARS-CoV-2 replication^8,10,22^. Acute tubular necrosis was the main renal lesion found in autopsies, since these cells express the ACE2 receptor^10,16,20,23^. This direct infection of renal cells was proposed as a mechanism of acute renal damage, given the characteristics of acute renal lesions found, such as extensive tubular epithelial vacuolization^10,22^.

#### Other changes

The studies also evidenced other alterations. In the liver tissue, the main findings were as follows: fibrosis, steatosis, centrilobular congestion, hepatomegaly, and coagulative necrosis mainly around the central veins, a condition associated with lobular hepatitis triggered by some drugs. However, these findings could be due to the patient’s past pathological history, as no specific histopathological correlation has been demonstrated for direct lesions caused by SARS-CoV-2, although viral particles have been detected^13^. These events are due to mechanisms such as cytokine storm, hypoxia, hypovolemia, and aggravation of chronic lesions of preexisting conditions. Dominic et al. speculated that ischemic liver lesions may indicate the presence of hepatic vascular thrombosis^14,16,20^.

In the central nervous system, a mild inflammatory infiltrate of T lymphocytes was observed around the vessels, and ischemic alterations were also found in neurons of the cortex and white matter. Moderate to intense activation of microglia was observed as the most prominent pathological feature^11,14,16^.

Digestive and pancreatic alterations were rarely noted. However, mild lymphocytic inflammatory infiltrate was observed in the digestive system and hemorrhagic pancreatitis^11,23^.

In the seminiferous tubules, especially in the Sertoli cells, vacuolization and cytoplasmic rarefaction were observed^19^. Another alteration found was the loss and desquamation of the intratubular cells in the lumens of the seminiferous tubules. Edema and inflammatory lymphocytic infiltrates were found in the interstitium.

## CONCLUSIONS

Autopsy plays an enormous role in the study of the pathogenesis of COVID-19 and contributes to the design of therapeutic plans as well as to the prognostic definition. There are still many limitations in the existing studies, both in relation to design and sample size. Thus, this review represents a stimulus for future studies that confirm the relationship between the infection by COVID-19 and possible systemic findings. We also highlighted the association between thrombotic events evidenced in various studies and infection by SARS-CoV-2, which is consistent with the published literature.
